# Computational Models of Emotion Inference in Theory of Mind: A Review and Roadmap

**DOI:** 10.1111/tops.12371

**Published:** 2018-07-31

**Authors:** Desmond C. Ong, Jamil Zaki, Noah D. Goodman

**Affiliations:** ^1^ A*STAR Artificial Intelligence Initiative Agency for Science, Technology and Research (A*STAR); ^2^ Institute of High Performance Computing Agency for Science, Technology and Research (A*STAR); ^3^ Department of Psychology Stanford University; ^4^ Department of Computer Science Stanford University

**Keywords:** Emotion, Affective cognition, Inference, Theory of mind

## Abstract

Research on social cognition has fruitfully applied computational modeling approaches to explain how observers understand and reason about others’ mental states. By contrast, there has been less work on modeling observers’ understanding of *emotional* states. We propose an intuitive theory framework to studying *affective cognition*—how humans reason about emotions—and derive a taxonomy of inferences within affective cognition. Using this taxonomy, we review formal computational modeling work on such inferences, including causal reasoning about how others react to events, reasoning about unseen causes of emotions, reasoning with multiple cues, as well as reasoning from emotions to other mental states. In addition, we provide a roadmap for future research by charting out inferences—such as hypothetical and counterfactual reasoning about emotions—that are ripe for future computational modeling work. This framework proposes unifying these various types of reasoning as Bayesian inference within a common “intuitive Theory of Emotion.” Finally, we end with a discussion of important theoretical and methodological challenges that lie ahead in modeling affective cognition.

## Abstract 2

An important, but relatively neglected, aspect of human theory of mind is emotion inference: understanding how and why a person feels a certain why is central to reasoning about their beliefs, desires and plans. The authors review recent work that has begun to unveil the structure and determinants of emotion inference, organizing them within a unified probabilistic framework.

## 1 Introduction

Recent developments in computational cognitive modeling have allowed researchers to specify and test precise hypotheses about how people make inferences about their social world. These include inferences about what others desire and believe about the world (e.g., Baker, Jara‐Ettinger, Saxe, & Tenenbaum, 2017; Goodman et al., 2006), what others mean when they use language to communicate (Frank & Goodman, 2012; Goodman & Frank, 2016; Goodman & Stuhlmüller, 2013), and what future decisions others might make (Jara‐Ettinger, Gweon, Schulz, & Tenenbaum, 2016; Jern & Kemp, 2015). These recent developments comprise the focus of the current special issue. However, these models have often neglected how people reason about one of the most essential elements of human psychology: *emotions*.

Emotions play a central role in our social lives. They signal people's immediate reactions to events in the world (e.g., Ellsworth & Scherer, 2003) and cause many behaviors—both intentional and unintentional (Lerner, Li, Valdesolo, & Kassam, 2015; Loewenstein & Lerner, 2003). Given the importance of emotions in social interactions, it is hardly surprising that people are naturally attuned to perceiving and understanding emotions in those around them (Harris, 1989; Zaki & Ochsner, 2011).

In this paper, we consider the present and future of computational modeling in understanding how people reason about emotional states—what we term *affective cognition* (Ong, Zaki, & Goodman, 2015). We adopt an intuitive theory framework to the study of affective cognition, using which we derive a taxonomy of affective cognitive inferences (in the spirit of Adelson & Bergen, 1991; Kemp & Jern, 2014, who, respectively, derived taxonomies of visual functions and inductive problems). This taxonomy encompasses a wide range of lay reasoning about emotional states, such as using observed behavior or situation contexts (or combinations thereof) to infer emotions and other mental states (e.g., de Melo, Carnevale, Read, & Gratch, 2014; Wu, Baker, Tenenbaum, & Schulz, 2018; Zaki, 2013). Finally, we provide a roadmap for future research, by highlighting inferences that have yet to be studied computationally, and by discussing important theoretical and methodological challenges that lie ahead.

## 2 Laying out an intuitive theory of emotions

People have rich intuitive theories of how others around them think and behave, allowing them to infer others’ motivations and explain others’ behavior (e.g., Gopnik & Meltzoff, 1997; Heider, 1958; Ross, 1977). An intuitive theory of other people consists of, first, a structured ontology of concepts—for example, *personality*,* goals*,* behavior*—and, second, the causal relationships relating these concepts (Gerstenberg & Tenenbaum, 2017). These intuitive theories allow laypeople to make sense of others around them, in a similar fashion to how scientific theories allow scientists to explain the physical world (Carey, 2009; Wellman & Gelman, 1992).

People also possess a rich intuitive theory of *emotions* that comprises conceptual knowledge about different emotional states (e.g., *anger*,* happiness*) and how they are related to their causes and effects. People (“observers”) use their intuitive theory of emotion to reason about the emotional states of others (“agents”) around them, and thereby decide how best to respond in social situations. Importantly, these intuitive theories comprise the observer's *beliefs* about how others’ emotions work, which depend on the observer's past history and their subjective beliefs. Although the observer's beliefs may not necessarily reflect the reality of how emotions “actually work,” these beliefs nevertheless form the basis for how the observer understands and interacts with those around her (e.g., Gopnik & Meltzoff, 1997; Ross, 1977).

**Figure 1:**
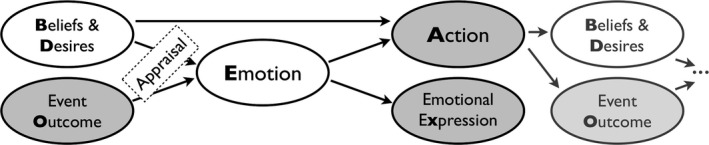
A model of an intuitive theory of emotion, unifying ideas from de Melo et al. (2014), Ong, Zaki, et al. (2015), Saxe and Houlihan (2017), and Wu et al. (2018). We use standard graphical model notation: Shaded circles represent observable variables, whereas unshaded circles represent latent variables. We render variables at the subsequent “time‐step” translucent. Arrows represent a directed causal relationship, and bolded letters denote the abbreviations used in equations. The observer applies a “**third‐person appraisal**” process to reason about how (a) the **outcome** of an event that an agent experiences, and (b) the agent's mental states (**beliefs** and **desires**), together result in the agent experiencing **emotions**. The agent's emotions in turn cause the agent to display **emotional expressions** and take intentional **actions** that lead to new outcomes and updated mental states (and new emotions).

An intuitive theory of emotion contains two important types of causal relationships. The first connects emotions to their causes: What, in the observer's mind, causes an agent to feel an emotion? The second involves the effects of emotion: What, in the observer's mind, does an agent's emotion cause them to do? These components and their relationships are represented in Fig. 1.

### 2.1 Intuitive causes of emotions

People intuitively expect that others’ emotions arise as a reaction to motivationally salient events. In addition to the outcome of these events, people also use their knowledge about others’ mental states—such as others’ beliefs and desires—to reason about how others feel. This intuitive understanding of how event outcomes and mental states impact emotions emerges early in life (Gross & Ballif, 1991; Harris, 1989; Lagattuta, Wellman, & Flavell, 1997; Repacholi, Meltzoff, Hennings, & Ruba, 2016; Wu, Muentener, & Schulz, 2017): Toddlers and infants understand that an agent's display of happiness reflects a satisfied desire, while an agent's display of sadness reflects a thwarted goal (Repacholi & Gopnik, 1997; Skerry & Spelke, 2014; Wellman, Phillips, & Rodriguez, 2000). By preschool, children associate a surprised emotional expression with a mismatch between reality and the agent's prior beliefs about the world (Wellman & Banerjee, 1991; Wu & Schulz, 2017), and factor an agent’s expectations into attributions of the agent's emotions (Ong, Asaba, & Gweon, 2016). Adults similarly draw rich inferences from an agent's emotional reactions to their latent beliefs and desires (Scherer & Grandjean, 2008; Van Kleef, De Dreu, & Manstead, 2010; Wu et al., 2018). Thus, the intuitive theory of emotion relates an agent's mental states (specifically, their **beliefs** about the world and their **desires**), and the **outcome** of an event that the agent experiences, to the agent's **emotions** (Fig. 1).

How do people understand the way event outcomes and the agent's mental states give rise to emotions? One idea is that people may engage a similar reasoning process to how they themselves experience events firsthand. According to appraisal theories of emotion (Arnold, 1960; Ellsworth & Scherer, 2003; Moors, Ellsworth, Scherer, & Frijda, 2013; Ortony, Clore, & Collins, 1988; Smith & Lazarus, 1993), an agent's emotions arise from an evaluation (“*appraisal*”) of outcomes along various self‐relevant dimensions, such as whether the outcomes contribute to or detract from the agent's goals (“goal‐conduciveness”). If Sally judges her job offer as facilitating her career goals, she would likely feel happy.

Many researchers have proposed that laypeople perform a similar appraisal‐like process to reason about others’ emotions (de Melo et al., 2014; Ong, Zaki, et al., 2015; Seimer & Reisenzein, 2007; Skerry & Saxe, 2015; Wondra & Ellsworth, 2015; Wu et al., 2018; Van Kleef et al., 2010)—we term this process “**third‐person appraisal**.” That is, when an observer considers an agent experiencing an outcome, the observer first reduces the outcome to a small number of features *that the observer thinks is relevant to the agent*, and then uses that evaluation to judge the agent's emotions. For example, observers intuitively evaluate, from the agent's perspective, an outcome's goal‐conduciveness and whether the outcome was expected. If Bob learns that Sally just received glowing performance reviews, and knows of her goals of getting promoted, he would reason that she probably feels a positively valenced emotion. If Bob additionally knows that Sally did not expect the performance reviews, he might predict that she would also feel surprised. Appraisal is especially important from a computational standpoint. The situations that agents experience in daily life vary widely along an innumerable number of dimensions, and (the observer's theory of) an agent's appraisal reduces the complexities of everyday situations to a small set of relevant “features” that are important for the agent's well‐being. It is also important to note that the observer's appraisal on behalf of the agent may differ from the agent's own appraisal in two key ways. First, the observer's set of emotionally relevant features, encapsulated within their intuitive theory, may not be the same as the set of features that the agent themselves consider, such as when an observer brings her culturally held norms about emotions to assess an outcome experienced by an agent from a different culture. Second, the observer's third‐person appraisals depend on the observer's inferences of the agent's prior beliefs and desires, which may be different from the agent's true mental states. If Bob thought that Sally had known about her performance reviews beforehand, he would not have predicted her surprise.

These first few pieces of a model of the intuitive theory (see left half of Fig. 1), describe the observer's rich causal knowledge about how outcomes and the agent's mental states “cause” emotion. This reasoning from outcomes and mental states to emotions relies on a contextualized, third‐person appraisal process, which we will describe in greater detail later. We turn next to the right half of Fig. 1.

### 2.2 Intuitive effects of emotions

People also intuitively reason about what behaviors emotions cause. For one, emotional agents produce a wide variety of **emotional expressions**. Decades of scientific research have shown that people can reliably recognize emotions from facial expressions (Ekman, Friesen, & Ellsworth, 1972; Elfenbein & Ambady, 2002; Russell, Bachorowski, & Fernández‐Dols, 2003), body language (Dael, Mortillaro, & Scherer, 2012), and emotional vocalizations and other paralinguistic changes in speech (Banse & Scherer, 1996; Johnstone & Scherer, 2000; Scherer, 2003). Along with emotional displays, emotions also influence the intentional **actions** that the agent may take next (Lerner et al., 2015; Loewenstein & Lerner, 2003). Fear may produce a tendency to flee, anger may prompt someone to approach and confront a threat, and happiness might engender prosocial behavior (e.g., Frijda, Kuipers, & Ter Schure, 1989; Isen, 1987). People are intuitively sensitive to how emotions can influence others’ actions; for example, people use a partner's emotional displays to infer how cooperative they would be (Krumhuber et al., 2007; Levine, Barasch, Rand, Berman, & Small, 2018; Van Kleef et al., 2010). In the intuitive theory in Fig. 1, we have placed emotional expressions and intentional actions as downstream effects of emotion, reflecting the intuitive notion that emotions “cause” these two types of behavior. Both types of behavior are readily observable in daily life and provide crucial signals that allow observers to reason “backwards” to infer the agent's latent emotion.

## 3 A taxonomy of affective cognitive inferences within the intuitive theory

In the previous section, we laid out a model of an intuitive theory of emotion (Fig. 1) by considering how emotions are related to their two antecedent causes and their two consequent effects. Using this intuitive theory, we can derive a taxonomy of inferences via exhaustive enumeration (see Supporting Information). Treating the model in Fig. 1 as a Bayesian network, we first wrote out all the 47 possible inferences that have emotion as one of the variables—for example, *P*(*emotion*|*outcome*), inferring emotions from outcomes, or *P*(*expression*|*emotion*), reasoning about expressions from emotions. Next, we removed 26 inferences that reduced to simpler inferences based on conditional independence.^1^ Third, we classified the remaining 21 inferences into six categories. Finally, we added a seventh category to account for counterfactual inferences, such as inferring emotions if a given outcome had not occurred, *P*(*emotion*|**not** *outcome*). This is summarized in Table 1.

**Table 1:** A taxonomy of inferences within affective cognition, derived from the model in Fig. 1

Categories	Description	Inferences	Examples
Emotion recognition	Infer an agent's emotions from emotional expressions (facial expressions, body language, prosody), *or* from their actions.	*P*(*e*|*x*) *P*(*e*|*a*)	Given *smile*, Infer *P*(*happy*) Given *scold‐others*, Infer *P*(*anger*)
Third‐person appraisals	Reason “forward” about how an event would cause an agent to feel, given also their mental states.	*P*(*e*|*o*) *P*(*e*|*o*,*b*,*d*) *P*(*e*|*b*,*d*)	Given *lose‐wallet*, Infer *P*(*sad*)
Inferring causes of emotions	Reason “backwards” about the events that caused an agent's emotions.	*P*(*o*|*e*) *P*(*o*|*e*,*b*,*d*)	Given *sad*, Infer *P*(*lose‐wallet*)
Emotional cue integration	Given multiple, potentially conflicting cues (e.g., multiple behaviors and/or causes of emotion), combine them and reason about agent's emotions	e.g., *P*(*e*|*o*,*x*) *P*(*e*|*o*,*a*) *P*(*e*|*a*,*x*) *P*(*e*|*o*,*b*,*d*,*a*,*x*)	Given *smile* + *lose‐wallet*, Infer *P*(*happy*), *P*(*sad*), etc.
Reverse appraisal	Given an event and an agent's emotions, reason backwards to mental states like beliefs and desires	*P*(*b*,*d*|*e*,*o*) *P*(*b*,*d*|*e*)	Given: *receive‐gift* + *surprise* + *happy*, Infer *Gift‐Unexpected* + *Gift‐Desired*
Predictions (hypothetical reasoning)	Given an agent's emotions, predict subsequent behavior. Or, given a (hypothetical) situation, predict the agent's emotions.	*P*(*a*|*e*) *P*(*x*|*e*)	If *anger*, predict *P*(*scold‐others*)
Counterfactual reasoning (and explanations)	Given a state of the world and an agent's emotions, reason about emotions or behavior in counterfactual states of the world. This also allows explanations of emotions or behavior in terms of their causes.	e.g., *P*(*e*|**not** *o*) *P*(*a*|**not** *e*)	Given: “*lose‐wallet* and *sad*,” reason that: “**if** *not‐lose‐wallet*,** then** *not‐sad*” or, reason that: “*sad * **because** *lose‐wallet*”

*Note*.

*P*(*X*) denotes the probability of *X* occurring. The lists of inferences presented for each category are exhaustive (given our derivation), except for the cue integration and counterfactual reasoning categories.

### 3.1 Emotion recognition

Perhaps the easiest category of lay inference is inferring someone's emotional state from their emotional expressions, often called **emotion recognition** (e.g., Elfenbein & Ambady, 2002). The deceptive ease by which laypeople “read” emotional expressions has led many researchers to assume perfect emotion recognition in their models, without modeling this process as an inference in its own right (e.g., de Melo et al., 2014; Wu et al., 2018).

In reality, laypeople's emotion recognition is not perfectly accurate, nor is it homogenous across all observers. First, such inferences are heavily dependent on context: Both intense positive and negative events may sometimes produce perceptually similar facial expressions, making it difficult for lay observers to accurately infer emotions solely from expressions (Aviezer, Trope, & Todorov, 2012; Carroll & Russell, 1996; Wenzler, Levine, van Dick, Oertel‐Knöchel, & Aviezer, 2016). Second, people from different cultures differ systematically in how they perceive emotions from facial expressions (Gendron, Roberson, van der Vyver, & Barrett, 2014; Jack, Blais, Scheepers, Schyns, & Caldara, 2009; Russell, 1994; Yuki, Maddux, & Masuda, 2007). Such inferences rely on lay observers’ intuitive theories, which in turn depend on their cultural background and past history (Jack, Caldara, & Schyns, 2012). Indeed, these cultural differences have been found to impact even how observers attend to and use low‐level facial cues when judging emotions; for example, East Asian participants tend to fixate longer on the eye region than Western participants (Jack et al., 2009; Yuki et al., 2007).

More recently, researchers have started building formal computational models of how observers infer emotions from low‐level features of facial expressions (Delis et al., 2016; Jack et al., 2012; Martinez & Du, 2012) and body language (Schindler, Van Gool, & de Gelder, 2008). Using conditional probability notation, modeling emotion recognition corresponds to estimating the probability of a latent emotion *e* given an emotional expression *x*, or *P*(*e*|*x*). The general recipe followed by these studies first requires some representation of the emotional expression—for example, a popular representation for facial expressions is the Facial Action Coding System (Ekman & Friesen, 1978). Next, one measures how human observers classify stimuli into pre‐defined emotion categories, that is, empirically measuring *P*(*e*|*x*) for say, the six “basic” emotion categories. Finally, one then models the emotion recognition process *P*(*e*|*x*) by training classifiers using sophisticated machine learning techniques. Such work is relatively new, and there is still much more to be done in modeling human emotion recognition from expressions and also from other behavior. This will provide a crucial foundation for many of the more complex inferences we discuss below.

### 3.2 Third‐person appraisal

Computational approaches allow researchers to quantitatively model how people reason about an agent's response to an experienced event. This involves, as mentioned, a **third‐person appraisal** process that reduces the outcome that an agent experiences into a small set of features that the observer thinks is important to the agent's emotions (e.g., Skerry & Saxe, 2015). To study this appraisal process in a controlled scenario, we designed a gambling scenario where we could parametrically vary the payoff amounts and probabilities, and we measured how people's judgments of an agent's emotions after the gamble depended upon these gamble features (Ong, Zaki, et al., 2015). We found that participants’ emotion judgments depended not only on the amount won, but how much the agent won compared to the expected value of the gamble, often called a *prediction error* (e.g., Sutton & Barto, 1998), suggesting that participants evaluated the agent's earnings relative to a reference point, rather than judging their earnings in absolute terms. We also found that lay participants judged agents to react more strongly to negative prediction errors than to positive prediction errors. These third‐person findings are strikingly consistent with influential scientific theories of first‐person utility, especially notions of reference‐dependent utility and loss aversion (Kahneman & Tversky, 1979; Kermer, Driver‐Linn, Wilson, & Gilbert, 2006; Mellers, Schwartz, Ho, & Ritov, 1997). Finally, participants believed that agents would feel worse if they came very “close” to winning the next higher outcome, compared to if agents were not close to winning the better outcome (Ong, Goodman, & Zaki, 2015). That is, participants’ intuitive theories were also sensitive to the “closeness” of alternative outcomes (Gleicher et al., 1990; Johnson, 1986; Kahneman & Tversky, 1982), which have yet to feature in first‐person appraisal theories.

The situation features we studied—prediction errors and the “closeness” of the outcome to alternative outcomes—are well‐defined within our experimental scenario, but much more work is needed to study third‐person appraisals in more complex, real‐world situations. The main challenges for future research would be defining a comprehensive representation for the many appraisal dimensions—using lists from first‐person appraisal theory (e.g., Ellsworth & Scherer, 2003; Ortony et al., 1988), or via “data‐driven” approaches like representational similarity analyses (Skerry & Saxe, 2015)—and modeling how people map these appraisal dimensions onto emotions. There also exists many technical challenges in automatically extracting these appraisal dimensions from naturalistic sources like visual scenes or vignette descriptions, themselves problems at the forefront of modern computer science.

### 3.3 Inferring causes of emotions

Given an observed emotion, people can also **reason “backwards” about potential causes of that emotion**. Upon encountering a sad friend, most people would start generating potential causes of the friend's sadness, such as possibly failing an exam. Intuitively, these possibilities vary in how likely they were to have happened: Failing an exam is more likely when one is a student, but less likely after one has graduated. They also range in how likely they were to have caused the observed emotion: A hysterically bawling friend probably experienced something worse than a failed exam. People intuitively agree that a potential cause with a high probability of occurring *and* a high probability of causing the observed emotion is a more likely candidate than either one with a lower probability of occurring or one with a lower causal strength.

One way to mathematically describe this reasoning is via Bayesian inference. First, we represent the third‐person appraisal process as *P*(*e*|*o*), a collection of probability distributions of emotion *e* given an outcome *o*.^2^ Then, by Bayes’s Rule, we can write the posterior probability *P*(*o*|*e*), the probability of outcome *o* given emotion *e*, as:

(1) P(o|e)∝P(e|o)P(o).

The posterior probability (e.g., *P*(*fail‐exam*|*sad*)) is proportional to both the likelihood of that outcome causing the emotion (e.g., how likely is it that failing an exam will cause one's friend to be sad, *P*(*sad*|*fail‐exam*)) and the prior probability of that outcome occurring (e.g., how likely is it that they had just failed an exam, *P*(*fail‐exam*)). We tested whether Eq. (1) provides a good model of participants’ inferences of the posterior in an experiment with the same gambling context described in the previous section. Participants observed the emotions that the agent ostensibly felt after playing a gamble, as well as the possible outcomes, and were tasked with inferring how likely it was that each of the outcomes occurring. We found that a model based on Bayes’s rule (Eq. (1)) accurately predicted participants’ judgments of the posterior probabilities of unseen gamble outcomes (Ong, Zaki, et al., 2015), lending support to the claim that laypeople's affective cognition relies on a coherent use of a rich intuitive theory. Indeed, recent evidence suggests that even infants are sensitive to these causal probabilities, actively searching for hidden causes when faced with an emotion that is improbable given the observed cause (Wu et al., 2017).

Inferring causes of emotions relies on first understanding the third‐person appraisal process, and so the challenges inherent in modeling the third‐person appraisal process also apply here. In addition, Eq. (1) requires the observer to consider a set of possible outcomes. One challenge for future researchers is to define the set of possible outcomes that laypeople intuitively shortlist and consider, as well as the prior probabilities that laypeople assign those outcomes. Estimating such knowledge is also a key problem faced when applying Bayesian models to other forms of cognition (e.g., causal induction; Griffiths, 2017).

### 3.4 Emotional cue integration

People have little difficulty reasoning about the emotions that result from experiencing situation outcomes (*P*(*e*|*o*)), or identifying the emotions that produce observed facial expressions (*P*(*e*|*x*)). In real life, however, people are often presented with *combinations* of multiple cues to an agent's emotions and have to reconcile these multiple cues, an issue of **emotional cue integration** (Ong, Zaki, et al., 2015; Skerry & Saxe, 2014; Zaki, 2013). Perhaps Alice knows that a friend has lost his job (a negative outcome), but sees a smile on his face (a positive expression). How does she make sense of this?

Scientists have long pondered the relative importance of situation contexts and emotional expressions to observers’ judgments of emotions, especially in cases where these cues conflict (e.g., Goodenough & Tinker, 1931). On the one hand, scholars have argued that facial expressions evolved to communicate, and hence are (always) veridical cues to an agent's emotion (e.g., Buck, 1994). Thus, since the jobless friend is smiling, she must be happy, because smiling evolved to communicate happiness, and she would not be smiling otherwise. On the other hand, a wealth of other studies show that laypeople sometimes weight the situational context and other behavioral cues over facial expressions when attributing emotions (Aviezer et al., 2008; Carroll & Russell, 1996; Kayyal, Widen, & Russell, 2015; see Ong, Zaki, et al., 2015, for a more detailed discussion). This impasse underscores the need for a precise theory of how different cues are weighted and integrated.

Within our framework, emotional cue integration is simply a “higher‐order” inference that relies on the simpler inferences discussed earlier. Given the causal model in Fig. 1, we can write out a precise and optimal formula for how an observer should integrate these different sources of information: For example, the probability of a latent emotion *e* given an observed outcome *o* and expression *x*,* P*(*e|o*,*x*), is given by the following equation:

(2) $$P\left(e|o,x\right)\propto \frac{P(e|o)P(e|x)}{P(e)}.$$

Note that this inference integrates both the probability of the emotion given the outcome, *P*(*e*|*o*), and the probability of the emotion given the facial expression, *P*(*e*|*x*), inferences we discussed earlier. Unlike prior work in resolving cue conflict, this model does not assume a priori that any one type of cue (e.g., facial expressions) is more “privileged,” but weighs the cues according to their reliability in predicting the agent's latent emotion. To verify this model, we ran a series of experiments where we presented participants with combinations of outcomes (the outcome of a gamble that the agent experienced) and agents’ emotional expressions (either a facial expression or verbal utterance). A Bayesian model based on Eq. (2) accurately predicted lay observers’ judgments of agents’ emotions given these multiple cues (Ong, Zaki, et al., 2015). This model predicts that observers will be sensitive to the reliabilities of cues in context, and it allows for observers to weigh outcomes, faces, body expressions, and other cues to differing amounts depending on the context.

Emotional cue integration is not limited to simple two‐cue combinations. Under this Bayesian framework, cue integration is any higher‐order inference that relies in part on single‐cue inferences like third‐person appraisal (*P*(*e*|*o*,*b*,*d*)) and emotion recognition (*P*(*e*|*x*), *P*(*e*|*a*)): Once one has models of these simpler inferences, one can derive inference from any combination of cues.

### 3.5 Reverse appraisal: Inferring mental states from emotions

People not only draw inferences about an agent's emotions but also **use emotions to reason backwards about an agent's appraisals and mental states** (e.g., Hareli & Hess, 2010; Scherer & Grandjean, 2008). In particular, emotional reactions provide information as to whether an agent's goals were met and whether the agent expected a given outcome. A positive/negative emotional expression may signal that the outcome was congruent/incongruent with an agent's desires, and a surprised expression might signal that the outcome was unexpected given the agent's beliefs. Comparing to the third‐person appraisal process that maps outcomes and mental states to emotions, this current piece of reasoning works in reverse, taking an outcome and observed expression to infer the agent's mental states—what de Melo et al. (2014) term “**reverse appraisal**.”

Extending the formalism in previous sections, given a model of the appraisal process from the outcome and the agent's beliefs and desires *P*(*e*|*o*,*b*,*d*), we can write an inference similar to Eq. (1) for the agent's beliefs and desires:

P(b,d|e,o)∝P(e|b,d,o)P(b,d).

This, however, requires an inference of the latent emotion. If, instead, what we actually observe is an emotional expression *x* and the outcome *o*, and we are only interested in the mental states, we can marginalize out the latent emotion:

(3) $$P\left(b,d|x,o\right)\propto P\left(b,d\right)\sum_{e}P\left(x|e\right)P\left(e|b,d,o\right).$$

Note that in the previous sections (e.g., Eqs. (1), (2)), the appraisal process was encapsulated in *P*(*e*|*o*); here, we explicitly represent the agent's beliefs and desires, so now we write the appraisal process as *P*(*e*|*b*,*d*,*o*). As discussed earlier, this process itself factors through quantities such as the prediction error of the outcome. In Eq. (3), the goal of the inference is the conditional probability of the agent's beliefs and desires; the agent's emotions are only an “intermediate” quantity that we can marginalize (or sum out). From right to left, the expression in Eq. (3) considers the product of (a) the probability of an emotion *e* being a result of a belief, desire, and outcome *P*(*e*|*b*,*d*,*o*) (i.e., the appraisal process) and (b) the probability of the emotion *e* resulting in the observed expression *P*(*x*|*e*). We then consider all possible emotions *e* by summing this product over all possible emotions (“marginalizing” out *e*). Finally, this term is multiplied with the prior probability of that combination of belief and desire, *P*(*b*,*d*).

To test if laypeople's inferences can be modeled by such a Bayesian model, Wu et al. (2018, experiment 3) constructed scenarios where agents performed an action (putting a white powder into a colleague's coffee), witnessed the resultant outcome (the agent's colleague living or dying), and displayed an emotional reaction (e.g., a *surprised* facial expression). Participants read about these sequences of events and provided judgments of the agent's beliefs (what the agent thought the powder was: *poison* or *sugar*) and desires (whether or not the agent wanted to harm her colleague). Wu et al. (2018) present a graphical model and an inference equation similar to Fig. 1 and Eq. (3). We note two key differences: First, their posterior has an additional conditional dependence on the agent's actions (i.e., *P*(*b*,*d*|*x*,*o*,*a*))—we chose not to add actions into Eq. (3) for simplicity. Second, they assumed perfect emotion recognition and hence did not explicitly model the latent emotion as an intermediate quantity between the outcome and the agent's expression. Their Bayesian model accurately tracked participants’ inferences of the agent's beliefs and desires, providing further evidence that people reason about others’ emotional and mental states using a coherent intuitive theory.

In addition, inferring others’ appraisals is also important in predicting their future behavior. For example, if someone chooses to cooperate with you on a task, and smiles after doing so, you may infer that she had intended to cooperate, liked the outcome, and will likely do so again. In contrast, if she frowned instead of smiled, your inferences and your subsequent behavior in response would be very different: Perhaps she regretted cooperating and might not cooperate next time (see also Levine et al., 2018). de Melo et al. (2014) modeled these inferences of appraisals from emotional expressions in a stylized social dilemma game where two players each chose between a mutually beneficial option and a selfish option that benefitted oneself at the expense of one's partner. Some participants played against a partner who smiled after mutual cooperation, whereas other participants saw their partner frown after mutual cooperation. de Melo et al. (2014) proposed and found support for a “reverse appraisal” model whereby participants inferred either a cooperative or competitive intent from their partner's emotional expressions, and these inferences mediated participants’ subsequent behavior in future interactions.

Mental and emotional states are intimately related, and here we discussed modeling inferences of mental states from emotional information. Such work is crucial to integrate emotions into existing models of Theory of Mind (e.g., Baker, Saxe, & Tenenbaum, 2009; Baker et al., 2017). There exist many open questions that arise from such integration of emotions and mental states: For example, there are many complex interactions between desires and emotions: on the one hand, people have goals to regulate emotions, but conversely, emotions themselves influence how people prioritize their existing goals. How do laypeople reason about these, and how can researchers fit them both into a causal model? These are important psychological questions that should be addressed in future models of social and affective cognition.

### 3.6 Predictions (hypothetical reasoning)

Lay reasoning about others’ emotions is not limited to the here and now; people can reason about others’ potential behavior given a future or hypothetical emotion (*P*(*x*|*e*), *P*(*a*|*e*)). Reasoning hypothetically involves using one's intuitive theory to reason about novel scenarios that could be in the possible future, or merely in the realm of imagination. Planning a romantic marriage proposal involves incessant simulation of possible worlds and emotions in those worlds. Indeed, experiments that prompt participants for emotion attributions to a fictional character in a vignette or cartoon all invoke hypothetical reasoning, and the ease with which people engage in hypothetical reasoning is crucial to their enjoyment of film and fiction.

Hypothetical reasoning can be modeled by simply allowing the inferred variables to be hypothetical, for example, estimating the behavior given a hypothetical emotion *P*(*a*|*e*). Within Bayesian modeling, this can be operationalized as sampling “posterior predictives” under the model (i.e., conditional predictions of new data), and it has been applied to model lay predictions in other domains (Griffiths & Tenenbaum, 2006), but not yet in affective cognition. Future work should focus on applying such techniques to model laypeople's predictions of (hypothetical) emotions. Such work has important implications for designing computational agents that have to simulate and forecast their human user's emotions and behavior.

### 3.7 Counterfactuals reasoning (and explanations)

The final category of inferences in our taxonomy is **counterfactual reasoning** about emotions. While the previous few sections have touched upon reasoning about emotions given a state of the world, people can also reason about others’ emotions in states of the world that are different from the existing reality (Gleicher et al., 1990; Johnson, 1986). Indeed, emotions like *regret* are characterized by counterfactual reasoning (Beck & Crilly, 2009). Counterfactual thinking, like hypothetical reasoning, relies on a rich causal model that the observer can mentally manipulate to reason about other possible worlds (Byrne, 2002; Ross, 1997). Would Carol not be sad if she had not lost her wallet (*P*(*sad*|**not** *lose‐wallet*))? Would Charlie have been less curt if he had not just lost his job?

Reasoning with counterfactuals also allows observers to provide explanations (Halpern & Pearl, 2005; Lagnado, Gerstenberg, & Zultan, 2013) for others’ emotions and behavior (Malle, 1999, 2011). Explanation within affective cognition involves assigning causality of an emotion or a behavior to their possible causes (Böhm & Pfister, 2015; Ong, Zaki, & Goodman, 2016). Was Tim's disappointment after his speech due to his stuttering or the difficult questions he received after (or perhaps, both)? Explanations are important in choosing interventions to regulate the agent's emotions. If one thinks that Tim felt that his talk had not gone well because of the difficult questions he received, one might try helping Tim reappraise the difficult questions as a signal of engagement, not disdain, from the audience. Forming explanations and identifying the causes of behavior is also important in assigning responsibility, as in moral or legal judgments (e.g., Alicke, Mandel, Hilton, Gerstenberg, & Lagnado, 2015), especially in legal systems with lay juries.

Although there has not yet been any work in computationally modeling counterfactual reasoning in affective cognition, the use of probabilistic causal models to represent the intuitive theory of emotions should allow an immediate application of existing techniques (e.g., Pearl, 2001, 2009). Similar probabilistic models have recently been used in modeling lay counterfactual reasoning in other domains (Lucas & Kemp, 2015; Oaksford & Chater, 2010). We anticipate that future research will soon apply formal techniques to model counterfactual reasoning in affective cognition.

## 4 Discussion

In this paper we have outlined a framework for understanding affective cognition as inference within an intuitive theory of emotion. We derived a taxonomy of inferences (Table 1), and we have discussed these inferences both with respect to recent work in computationally modeling affective cognition, as well as future challenges for modeling each of these inferences. Importantly, this unified framework allows us to describe all the reasoning in Table 1 using the same general principles (i.e., Bayesian inference) applied to different “components” of the causal model in Fig. 1. In other words, how people reason from outcomes to emotion, or infer emotion from expressions, can all be modeled with the same domain‐general inference machinery, under a common Bayesian “Theory of Emotion” (Ong, Zaki, et al., 2015; Saxe & Houlihan, 2017).

Throughout the paper, we have posed affective cognition as a “*computational‐level*” problem (Marr, 1982) and have focused on reviewing probabilistic approaches, which offer a natural solution to such inferential problems (e.g., Griffiths, Kemp, & Tenenbaum, 2008; Oaksford & Chater, 2007). The Bayesian approaches in the studies described here (de Melo et al., 2014; Ong, Zaki, et al., 2015; Saxe & Houlihan, 2017; Wu et al., 2018) share more commonalities than differences, and what differences exist lie mainly in which variables the different sets of authors chose to prioritize or simplify out. Indeed, Fig. 1 results from our efforts to unify the common ideas from these studies—for instance, every study mentioned the crucial role of the third‐person appraisal process and its causal link to emotion. We think that this computational Bayesian framework offers a principled approach to unifying the inferences discussed, but it can also be supplemented by alternative, non‐Bayesian approaches. For example, perhaps the complex expression‐to‐emotion mapping might be best modeled with neural‐network approaches (e.g., Schindler et al., 2008). In addition, process models and neuroscientific approaches (e.g., Adolphs, 2002) may shed more light on Marr (1982)'s *algorithmic* and *implementation* levels of analysis. levels of analysis.

In our view, the biggest challenge ahead for models of affective cognition is finding suitable computational representations, especially of emotions: What is the space of emotions in the intuitive theory? Clearly, binary or multinomial (“angry” vs. not) labeling is insufficiently comprehensive, although widely used in many classification tasks (like sentiment analysis). Should researchers then represent emotions in some high‐dimensional space, and if so, what are those dimensions (Barrett & Russell, 1998; Mattek, Wolford, & Whalen, 2017)? How should mixed emotions be represented? Defining the representation space is a crucial prerequisite for probabilistic modeling, enabling sampling from and marginalizing over the space of emotions (as in Eq. (3)).

But even a vector in some high‐dimensional space may still be inadequate. Intuitively, the three scenarios (a) “John is angry,” (b) “John is angry at receiving a negative outcome,” and (c) “John is angry at the unfair process that led to the negative outcome” are all qualitatively different because of the appraisal that resulted in the emotions and the different behavioral consequences they connote. This insight demands a richer, *relational* representation of emotion that incorporates target‐ and event‐related information (minimally, “angry_at_X,” “sad_because_Y”), which current Bayesian graphical models do not support—although we think that modern incarnations like probabilistic programming languages may offer a solution (e.g., Goodman, 2013; see also Goodman & Frank, 2016). In our opinion, choosing a suitable representation for emotions (and appraisals) that captures the richness of lay affective cognition and that can be computed over efficiently may prove to be the largest hurdle to overcome.

Finally, we must stress that future work must prioritize modeling affective cognition on naturalistic data. The sparse stimuli (e.g., static facial expressions) and experimental scenarios in the studies discussed above are an important starting point, but they pale in comparison to the richness of everyday emotional experiences. It is important for future research to model how laypeople reason about the emotions of others in naturalistic contexts, such as watching someone in an unscripted monolog (e.g., Devlin, Zaki, Ong, & Gruber, 2014, 2016; Zaki, Bolger, & Ochsner, 2008) or dialog (e.g., Ickes, Stinson, Bissonnette, & Garcia, 1990). Modeling naturalistic contexts brings its own set of challenges—for example, extracting spontaneous emotional expressions and representing the complex appraisals of naturalistic situations—but there is also great payoff. First, they afford real‐world tests of the validity of computational models developed on “clean” laboratory stimuli. Second, they will inform computational technologies that can reason about their users’ emotions in a human‐like manner (e.g., Picard, 1997).

In conclusion, we hope that the framework and taxonomy of inferences we present here provides a useful conceptualization of work in computational models of affective cognition. We have identified many important challenges that lie ahead for this nascent field of research, which we are confident will be addressed by future researchers. At a broader level, our approach integrates well with state‐of‐the‐art computational models in other domains of social cognition (Baker et al., 2017; Goodman & Frank, 2016; Goodman et al., 2006; Jara‐Ettinger et al., 2016)—this integration will be crucial for a cumulative science of social and affective reasoning.

## Conflict of interest

The authors declare no conflict of interest.

## Authors’ contribution

D.C.O., J. Z., and N. D. G. wrote the paper.

## Supporting information


**Appendix S1.** Calculations to support the derivation of the taxonomy of inferences in Table 1.

## Notes

1 For example, under the model, *P*(*outcome*|*emotion*,* expression*) reduces to *P*(*outcome*|*emotion*) as *outcomes* and *expressions* are independent given *emotions* (d‐separation, e.g., Koller & Friedman, 2009).

2 For the moment, we leave out the agent's beliefs and desires in the appraisal process. We add them later in the section on “reverse appraisal.”
